# Regionally Altered Immunosignals of Surfactant Protein-G, Vascular and Non-Vascular Elements of the Neurovascular Unit after Experimental Focal Cerebral Ischemia in Mice, Rats, and Sheep

**DOI:** 10.3390/ijms23115875

**Published:** 2022-05-24

**Authors:** Dominik Michalski, Willi Reimann, Emma Spielvogel, Bianca Mages, Bernd Biedermann, Henryk Barthel, Björn Nitzsche, Stefan Schob, Wolfgang Härtig

**Affiliations:** 1 Department of Neurology, University of Leipzig, Liebigstr. 20, 04103 Leipzig, Germany; willi.reimann@medizin.uni-leipzig.de (W.R.); emma-spielvogel@gmx.de (E.S.); 2 Paul Flechsig Institute for Brain Research, University of Leipzig, Liebigstr. 19, 04103 Leipzig, Germany; bernd.biedermann@medizin.uni-leipzig.de (B.B.); wolfgang.haertig@medizin.uni-leipzig.de (W.H.); 3 Institute of Anatomy, University of Leipzig, Liebigstr. 13, 04103 Leipzig, Germany; bianca.mages@medizin.uni-leipzig.de; 4 Department of Nuclear Medicine, University of Leipzig, Stephanstr. 11, 04103 Leipzig, Germany; henryk.barthel@medizin.uni-leipzig.de (H.B.); bjoern.nitzsche@medizin.uni-leipzig.de (B.N.); 5 Institute of Anatomy, Histology, and Embryology, Faculty of Veterinary Medicine, University of Leipzig, An den Tierkliniken 43, 04103 Leipzig, Germany; 6 Department of Neuroradiology, University of Halle, Ernst-Grube-Str. 40, 06120 Halle (Saale), Germany; stefan.schob@uk-halle.de

**Keywords:** surfactant protein-G, vasculature, collagen IV, neurovascular unit, stroke, focal cerebral ischemia

## Abstract

The surfactant protein-G (SP-G) has recently been discovered in the brain and linked to fluid balance regulations. Stroke is characterized by impaired vessel integrity, promoting water influx and edema formation. The neurovascular unit concept (NVU) has been generated to cover not only ischemic affections of neurons or vessels but also other regionally associated cells. This study provides the first spatio-temporal characterization of SP-G and NVU elements after experimental stroke. Immunofluorescence labeling was applied to explore SP-G, vascular and cellular markers in mice (4, 24, and 72 h of ischemia), rats (24 h of ischemia), and sheep (two weeks of ischemia). Extravasated albumin indicated vascular damage within ischemic areas. Quantifications revealed decreasing SP-G signals in the ischemia-affected neocortex and subcortex. Inverse immunosignals of SP-G and vascular elements existed throughout all models. Despite local associations between SP-G and the vasculature, a definite co-localization was not seen. Along with a decreased SP-G-immunoreactivity in ischemic areas, signals originating from neurons, glial elements, and the extracellular matrix exhibited morphological alterations or changed intensities. Collectively, this study revealed regional alterations of SP-G, vascular, and non-vascular NVU elements after ischemia, and may thus stimulate the discussion about the role of SP-G during stroke.

## 1. Introduction

Along with the development of pharmaceutical and endovascular recanalizing strategies and the establishment of specialized treatment centers, the clinical outcome of acute ischemic stroke improved significantly over the last decades [1]. Stroke nevertheless accounts for one of the three major causes of death and long-lasting disability worldwide [2].

Experimental research has focused on a better pathophysiological understanding of stroke as a pre-condition to conceptualize novel neuroprotective strategies [3]. The exploration of ischemic consequences to the brain thereby entailed the term neurovascular unit (NVU), which replaces the traditional neuro-centric perspective [4,5]. The most important feature of this modern concept is the consideration of regionally associated cell types in addition to neurons, i.e., microglia, astroglia, oligodendrocytes, and cells forming the vasculature, which are all affected in the setting of ischemia (e.g., [6,7,8,9]). A unique function of the NVU is the maintenance of the vasculature’s integrity and thus the blood-brain barrier [10]. Experimental studies have shown that an ischemic affection of the vasculature results in the extravasation of blood-sourced substances (e.g., [11]). Accordingly, clinical studies have described the development of space-occupying edema formation due to impaired integrity of the vasculature (e.g., [12]). In addition to an altered barrier function between blood and the brain’s parenchyma, the influx of cerebrospinal fluid was recognized as a significant factor for edema formation in stroke [13,14]. Further, the glymphatic system, representing a unique cleaning pathway that uses perivascular spaces to flush the brain’s interstitium was recently discussed to be involved in ischemia-associated edema formation [15,16,17]. In this context, a pivotal role in blood-brain barrier functionality and brain fluid homeostasis has been attributed to the aquaporin-4 (AQP4) water channel, which is typically located at the astroglial end-feet [18,19,20,21]. Neuroprotective approaches in stroke thus included supportive approaches to elements of the NVU, as a strategy to stabilize the vasculature’s integrity and prevent edema formation [22]. However, successful translation into a clinical application is still challenging [23].

Among the factors that were not yet tested regarding their neuroprotective potential, the group of surfactant proteins (SPs) could be of interest, as they might also impact the fluid balance in the brain. Four traditional SPs, i.e., SP-A, SP-B, SP-C, and SP-D, were initially discovered in the lung and found as part of the pulmonary surfactant, which comprises serum-derived proteins [24]. These findings suggest an actively regulated barrier between the blood and the alveoli in the lung, a situation that is comparable to the brain with its inherent barriers. Remarkably, in a study involving healthy subjects and patients with hydrocephalus, the levels of SP-C in the cerebrospinal fluid were correlated with radiologically detectable flow rates [25]. Further, the traditional SPs were identified in human brain tissue by immunohistochemistry and were closely associated with the ventricles [26]. These observations strongly support the view that SPs might be involved in the brain’s fluid balance. Around ten years ago, the family of SPs was extended by SP-G, which displays a chemical similarity to SP-B and SP-C. SP-G was hypothesized to exhibit particular rheological and surface-regulatory properties [27]. Recently, the regional association of SP-G and selected elements of the NVU was investigated in naive mice, which indicated a co-localization with neuronal subpopulations [28]. Further, a study applying immunohistochemistry in naive rats provided the first evidence for a vessel-associated localization of SP-G [29]. 

However, SP-G has not yet been investigated in the setting of stroke and its importance for vasculature’s integrity is not understood. Based on multiple immunofluorescence labeling, the present study thus explored spatio-temporal characteristics of SP-G and the associated vasculature after experimental focal cerebral ischemia. Emphasis was also given to spatial relationships between SP-G and non-vascular elements of the NVU. For translational issues, the study setup included three animal species and thus involved a filament-based model in mice, an embolic model in rats, and a coagulation-based model in sheep.

## 2. Results

### 2.1. SP-G and Vascular Elements in Ischemia-Affected Brain Areas in Mice

**Figure 1:**
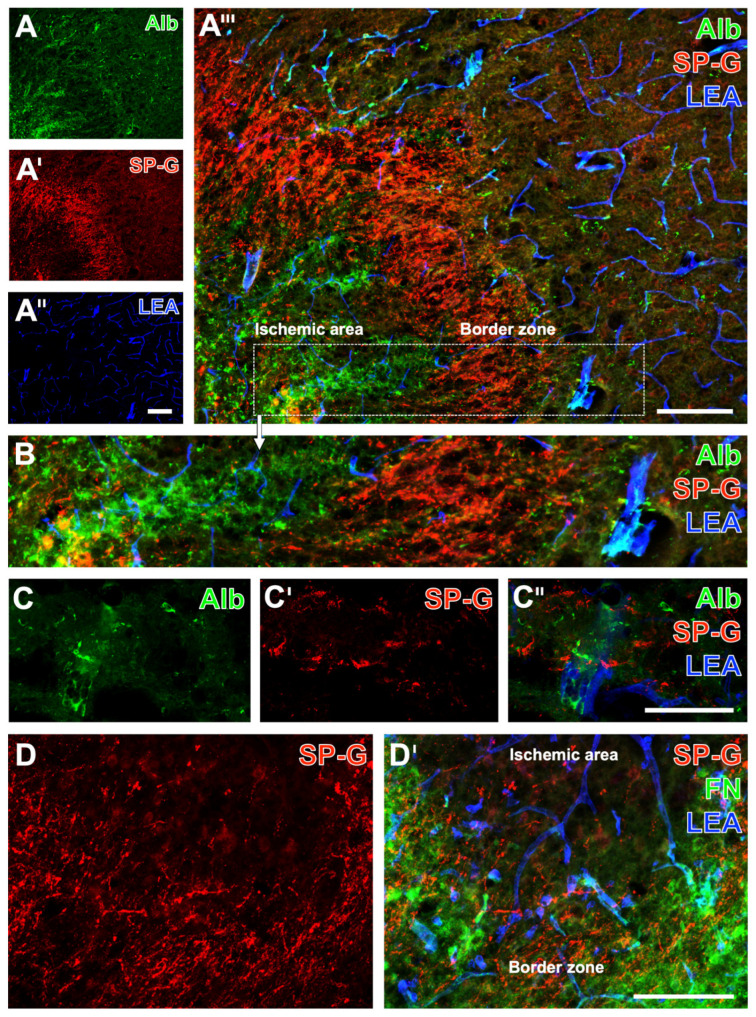
Surfactant protein-G (SP-G) and vascular endothelia in striatal ischemic areas of mice with impaired vasculature’s integrity indicated by extravasation of endogenous albumin into the parenchyma. Representative triple fluorescence labeling of albumin (Alb), SP-G (red), and lectin-based visualization of the vasculature (LEA) in mice 72 h after focal cerebral ischemia (**A**–**A’’’**), added by a higher magnification of the same labeling at the ischemic border zone (**B**). Exemplary combined detection of Alb, SP-G, and the vasculature (LEA) at a higher magnification captured by laser-scanning microscopy (**C**–**C’’**). Triple fluorescence labeling of SP-G, fibronectin (FN), and the vasculature (LEA) after 72 h of focal cerebral ischemia (**D**,**D’**). Scale bars: **A’’** (also valid for **A** and **A’**) = 100 μm, **A’’’** = 100 μm, **C’’** (also valid for **C** and **C’**) = 50 μm, **D’** (also valid for **D**) = 100 μm.

Experiments in mice indicated a relevant affection for the vasculature’s integrity in ischemic brain areas as visualized by extravasation of endogenous albumin into the brain’s parenchyma. As exemplarily shown in a mouse with 72 hours (h) of filament-based focal cerebral ischemia, the immunoreactivity (ir) for albumin was visually enhanced and appeared with coarse deposits in the ischemic striatum (Figure 1A,A’’’). Remarkably, the immunosignal of SP-G appeared to decrease in the area with maximum vascular affection, surrounded by a band with slightly increased SP-G-ir (bottom left in Figure 1A’,A’’’). However, simultaneous lectin-histochemical visualization of endothelia revealed a nearly constant distribution with a trend towards a rarefication in the ischemic area (Figure 1A’’,A’’’). Complementary patterns of immunosignals for SP-G and albumin became more apparent in the higher magnified border zone (Figure 1B). To explore spatial relationships between SP-G, albumin, and the vasculature in ischemic areas, laser-scanning microscopy at higher magnification was applied, showing SP-G-ir as local accumulations with a possible cellular association but also in a weaker and diffusely distributed formation, not overlapping with albumin or lectin-positive vascular elements (Figure 1C–C’’). Additional staining of fibronectin was performed to confirm the observed changes of SP-G-ir together with another marker sensitive to ischemia. Thereby, fluorescence labeling revealed both SP-G- and fibronectin-ir in the ischemic border zone, and SP-G was found to gradually decrease towards the region with the strongest ischemic affection (Figure 1D,D’). 

**Figure 2:**
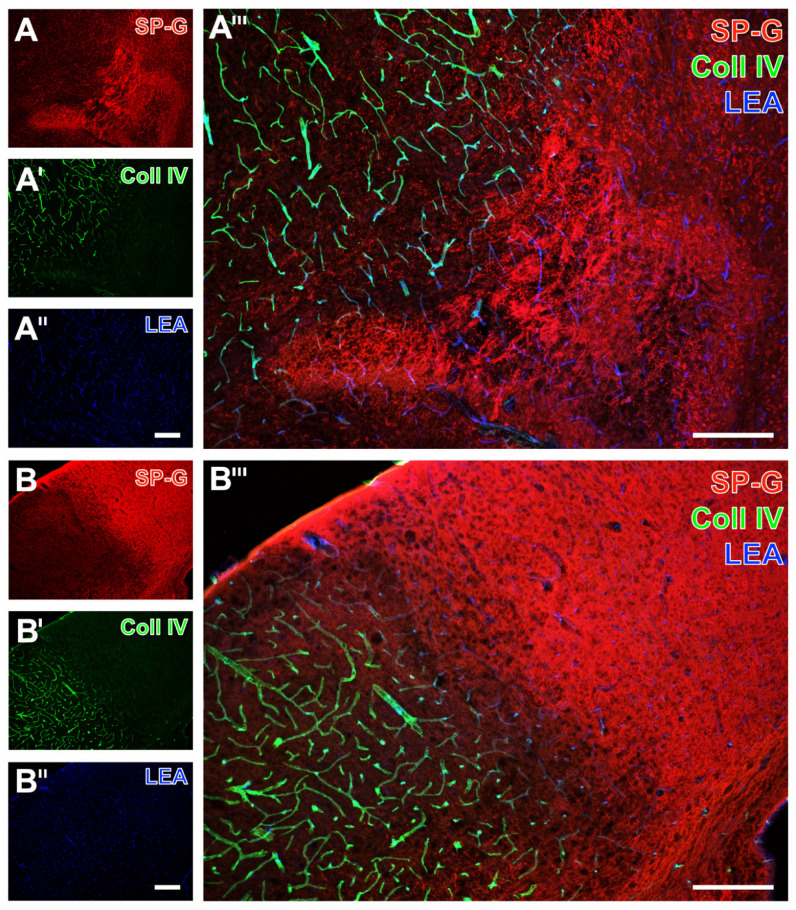
Regional associations of surfactant protein-G (SP-G) and vascular elements after 24 h of ischemia in mice depending on brain regions. Exemplary combined fluorescence labeling of SP-G, collagen IV (Coll IV), and lectin-based visualization of the vasculature (LEA) in the affected striatum (**A**–**A’’’**) and in the neocortex (**B**–**B’’’**) of mice 24 h after ischemia onset. Scale bars: **A’’** (also valid for **A** and **A’**) = 200 μm, **A’’’** = 200 μm, **B’’** (also valid for **B** and **B’**) = 200 μm, **B’’’** = 200 μm.

**Figure 3:**
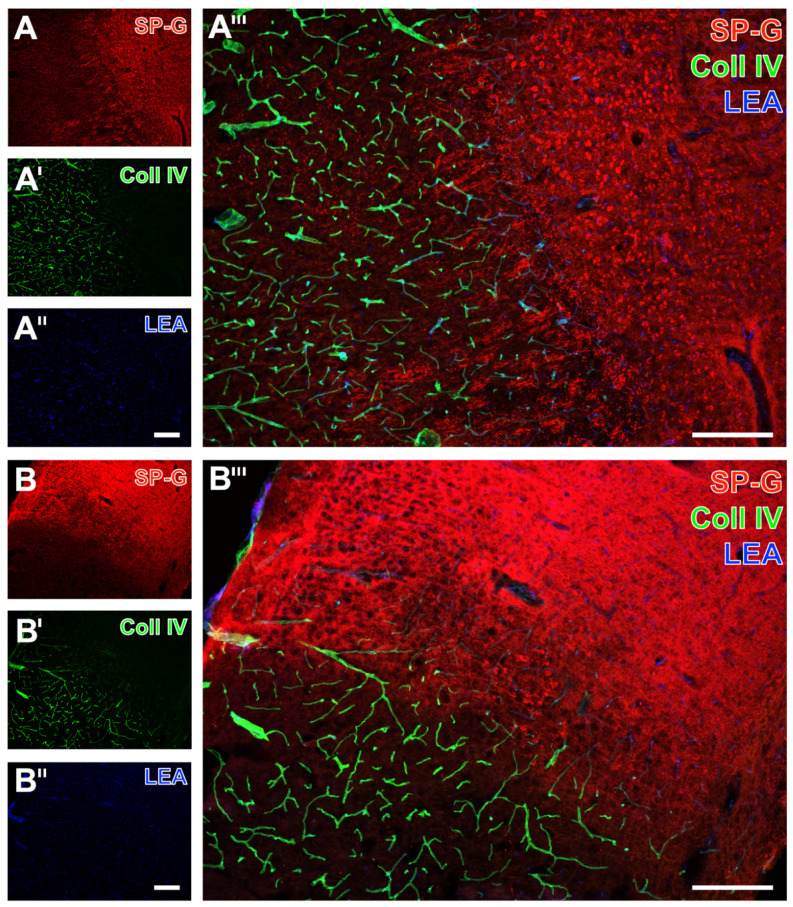
Regional associations of surfactant protein-G (SP-G) and vascular elements after 4 h of ischemia in mice depending on brain regions. Exemplary triple fluorescence labeling of SP-G, collagen IV (Coll IV), and vascular endothelia (LEA) in the affected striatum (**A**–**A’’’**) and in the neocortex (**B**–**B’’’**) of mice subjected to 4 h of focal cerebral ischemia. Scale bars: **A’’** (also valid for **A** and **A’**) = 200 μm, **A’’’** = 200 μm, **B’’** (also valid for **B** and **B’**) = 200 μm, **B’’’** = 200 μm.

Serial forebrain sections from mice were then applied to explore the temporal and regional characteristics of SP-G and associated vascular structures. For this purpose, SP-G immunolabeling was combined with two approaches for vessel staining: collagen IV immunolabeling of basement membranes and the detection of endothelia with tomato lectin (*Lycopersicon esculentum* agglutinin, LEA). In mice subjected to 24 h of focal cerebral ischemia, affected striatal regions were characterized by a decreased SP-G-ir in areas of maximum ischemia (top left in Figure 2A,A’’’), surrounded by a band with a slightly increased SP-G signal. In parallel, LEA was found evenly distributed with a trend towards a rarefied signal in the ischemic area (Figure 2A’’,A’’’), which concomitantly displayed enhanced collagen IV-ir (Figure 2A’,A’’’). Comparable staining patterns were found in the ischemia-affected neocortex with a decreased SP-G-ir and increased collagen IV-ir towards the ischemic area (Figure 2B,B’,B’’’). However, the band of a slightly increased SP-G immunosignal—consistently found in close vicinity to the maximum of striatal ischemia —was not detectable in the ischemia-affected neocortex. Mice subjected to 4 h of focal cerebral ischemia showed similar staining patterns of SP-G- and collagen IV-ir as mice with 24 h of ischemia. Thereby, SP-G-ir in the ischemia-affected striatum decreased while collagen IV-ir was enhanced (left part in Figure 3A,A’,A’’’). Similarly, the ischemia-affected neocortex presented lowered SP-G-ir and in parallel an increased collagen IV signal towards ischemia (bottom left in Figure 3B,B’,B’’’). In accordance with the findings at 24 h of ischemia, in mice subjected to 4 h of focal cerebral ischemia, a band-like increase of the SP-G signal in the spatial neighborhood to the area with maximum ischemia was detectable only in the striatum.

**Figure 4:**
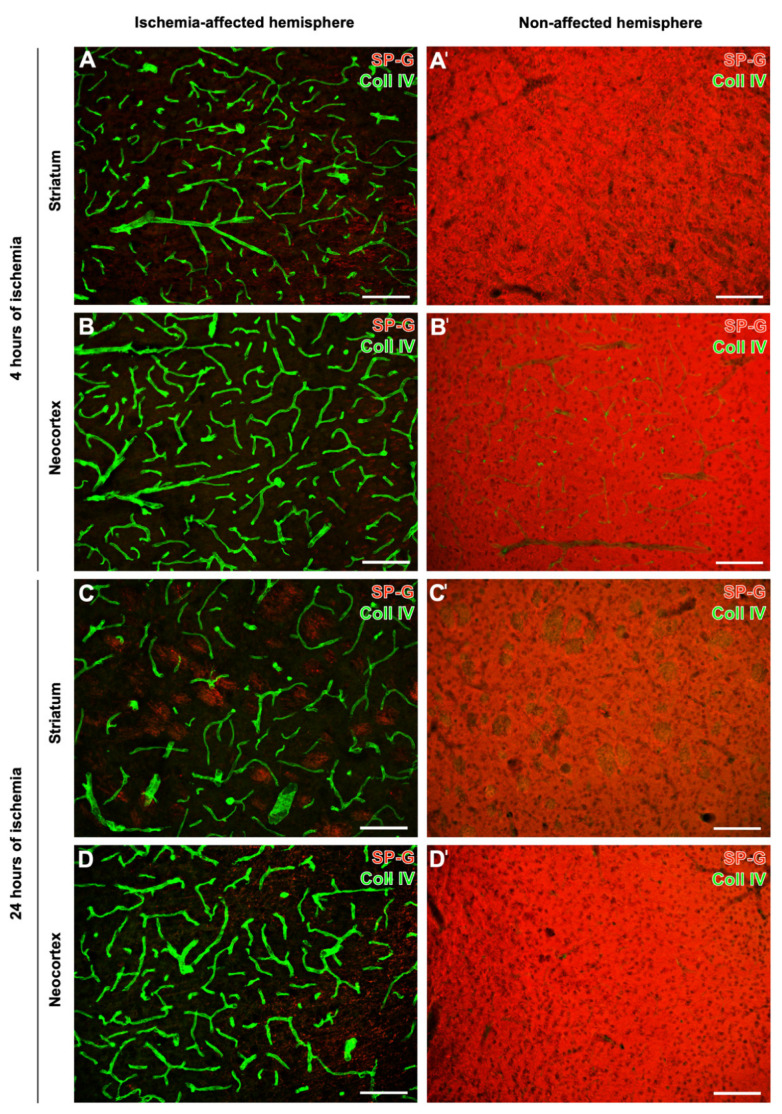
Surfactant protein-G (SP-G) and vascular elements after 4 h or 24 h of ischemia in mice depending on brain regions. Exemplary double fluorescence labeling of SP-G and collagen IV (Coll IV) in the affected and non-affected neocortex (**B**,**B’**,**D**,**D’**) and the affected and non-affected striatum (**A**,**A’**,**C**,**C’**) of mice subjected to 4 h or 24 h of focal cerebral ischemia. Scale bars: all = 100 μm.

In addition to regional characteristics of SP-G and the vasculature at the ischemic border zone of the affected neocortex and the striatum, a subset of analyses focused on inter-hemispheric differences. In mice subjected to 4 h or 24 h of focal cerebral ischemia, both ischemia-affected neocortex and striatum were characterized by a strong collagen IV-ir visualizing the vasculature, which co-occurred with a decreased signal of SP-G (Figure 4A–D). In contrast, the same regions on the contralateral, i.e., in the non-affected hemisphere were characterized by a strong SP-G signal, while only a few vascular elements displayed collagen IV staining (Figure 4A’–D’).

**Figure 5:**
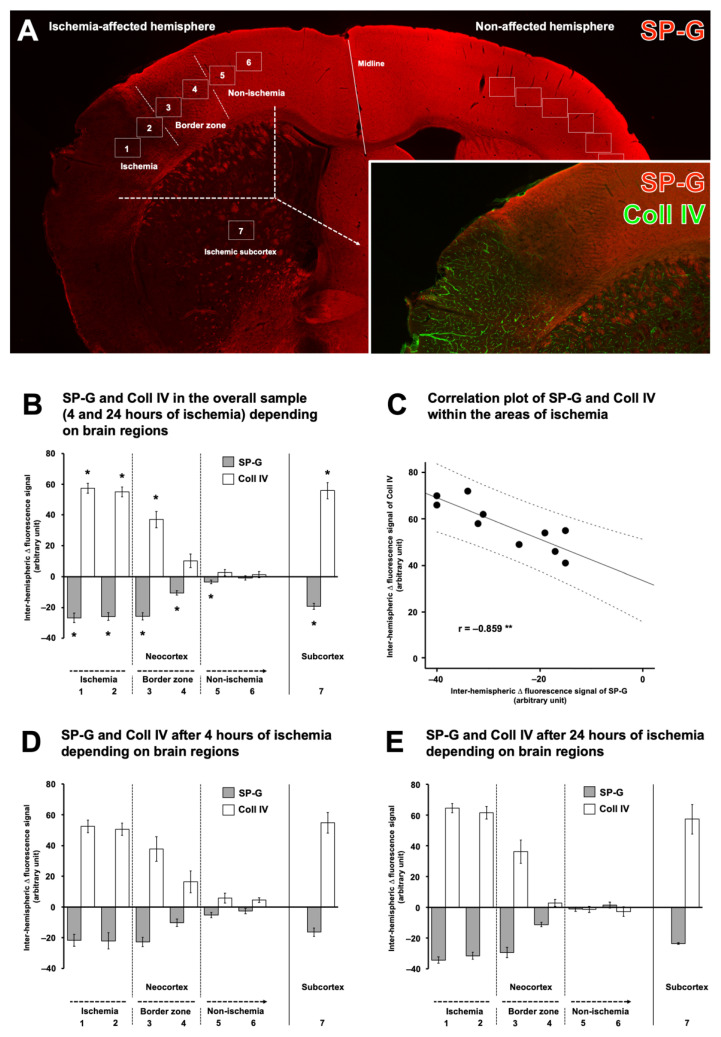
Quantification of immunosignals from surfactant protein-G (SP-G) and collagen (Coll) IV after 4 h and 24 h of ischemia in mice. Exemplary neocortical and subcortical arrangement of regions used for quantifications, based on serial sections from mice subjected to 4 h and 24 h of focal cerebral ischemia (**A**). Analyses in the overall sample (**B**), added by a calculated statistical association between immunosignals for SP-G and Coll IV (**C**). Data on subsets of the study sample, i.e., in mice subjected to 4 h of ischemia (**D**) and 24 h of ischemia (**E**). Analyses were based on 6 mice in the 4 h group and 5 mice in the 24 h group. Underlying animal numbers for each region ranged from 10 to 11 in the overall sample and 4 to 5 in the 24 h group, while 6 were available for each region in the 4 h group. Bars represent mean values as calculated by inter-hemispheric differences of immunofluorescence-based signals from SP-G and Coll IV. Added lines represent standard errors. Significance levels: *: *p* < 0.05, **: *p* < 0.01. r: Pearson correlation coefficient.

Quantitative analyses were performed to confirm the observed ischemia-associated alterations of SP-G and associated vascular elements. In addition to SP-G, collagen IV was chosen as the most robustly affected marker in the qualitative analyses. For this purpose, 7 regions of interest (ROIs) were positioned at the ischemia-affected hemisphere and were mirrored to the contralateral, i.e., non-affected hemisphere, to allow inter-hemispheric comparison (Figure 5A). In detail, 6 ROIs were used to capture alterations along the neocortex, ranging from the area with maximum ischemic affection along the border zone to the non-ischemic area. One additional ROI was used to capture alterations in the ischemia-affected subcortex, i.e., in the striatum. In the overall sample, a significantly decreased SPG immunosignal was found in the area with maximum ischemia compared to the non-affected hemisphere (*p* = 0.025 for each ROI). Further, a significantly decreased SP-G-ir was seen in the lateral border zone (*p* = 0.021). Towards the non-ischemic area, a significant but lesser decrease of the SP-G immunosignal was noted in the medial border zone (*p* = 0.021), whereas a slight but also significant decrease was observed at the lateral border of the non-affected area (*p* = 0.03) (Figure 5B). In the ischemia-affected subcortex, SP-G-ir was found to decrease significantly compared to the non-affected hemisphere (*p* = 0.025). Notably, inverse patterns for the immunosignals of collagen IV were found along the ischemia-affected neocortex and the striatum. In detail, immunosignals of collagen IV were found to increase significantly in the area with maximum ischemia (*p* = 0.03 for each ROI), while a gradual weaker increase was detected in the lateral border zone (*p* = 0.021). In the ischemia-affected subcortex, the immunosignal of collagen IV was also significantly increased (*p* = 0.03).

To explore the statistical relationship between inter-hemispheric differences of SP-G and collagen IV, a Pearson correlation was calculated (Figure 5C). Thereby, the negative correlation coefficient confirmed the observation of inversely associated signals of SP-G and collagen IV, which means that an increase of the collagen IV-ir concomitantly occurred with a decrease of SP-G-ir (*p* = 0.001). A subset of calculations addressed the immunosignals of SP-G and collagen IV along the ischemia-affected neocortex and the striatum depending on the period of ischemia, i.e., 4 h and 24 h. Comparable patterns were observed for both time points with a gradual decrease of the SP-G immunosignal and an increase of the collagen IV signal, especially in the neocortical area with maximum ischemia and the ischemic subcortex (Figure 5D,E). However, statistical significance was not achieved at the level of any inter-hemispheric comparisons (*p*-values ranged from 0.182 to 0.668 for SP-G and from 0.196 to 1.0 for collagen IV), which is likely due to the limited sample size in these sub-analyses.

### 2.2. Regional Characteristics of SP-G and Neurovascular Unit Elements after Ischemia

After characterizing alterations of SP-G and the associated vasculature due to focal cerebral ischemia, further analyses focused on spatial relationships within the ischemia-affected NVU. For this purpose, selected forebrain sections were used for multiple immunofluorescence labeling, including Neuronal Nuclei (NeuN) as a marker for neurons, 2′,3′-cyclic nucleotide phosphodiesterase (CNP) for oligodendrocytes, ionized calcium-binding adapter molecule-1 (Iba) for microglia, as well as glial fibrillary acidic protein (GFAP) and AQP4 for astroglial components.

**Figure 6:**
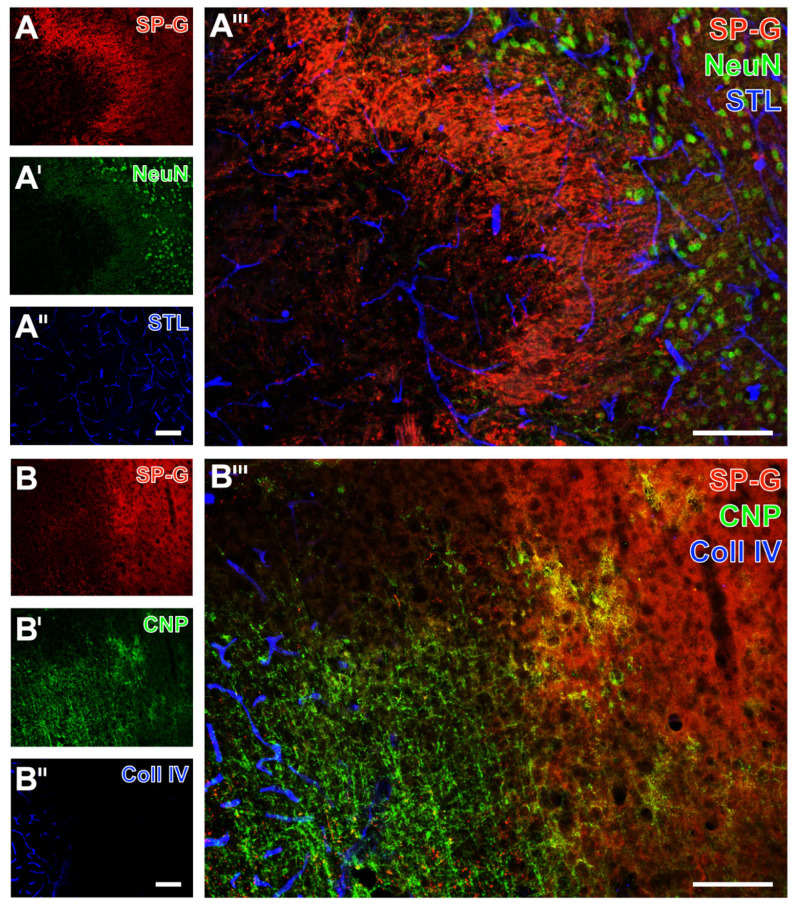
Spatial relationships between surfactant protein-G (SP-G), vascular elements, neurons, and oligodendrocytes after focal cerebral ischemia in mice. Exemplary triple fluorescence labeling of SP-G, Neuronal Nuclei (NeuN) for neurons, and the vasculature with *Solanum tuberosum* lectin (STL) in the affected striatum of a mouse subjected to 24 h of focal cerebral ischemia (**A**–**A’’’**). Representative triple immunofluorescence labeling of SP-G, 2′,3′-cyclic nucleotide phosphodiesterase (CNP) for oligodendrocytes and collagen (Coll) IV for the vasculature in the affected neocortex of a mouse 72 h after ischemia onset (**B**–**B’’’**). Scale bars: **A’’** (also valid for **A** and **A’**) = 100 μm, **A’’’** = 100 μm, **B’’** (also valid for **B** and **B’**) = 100 μm, **B’’’** = 100 μm.

**Figure 7:**
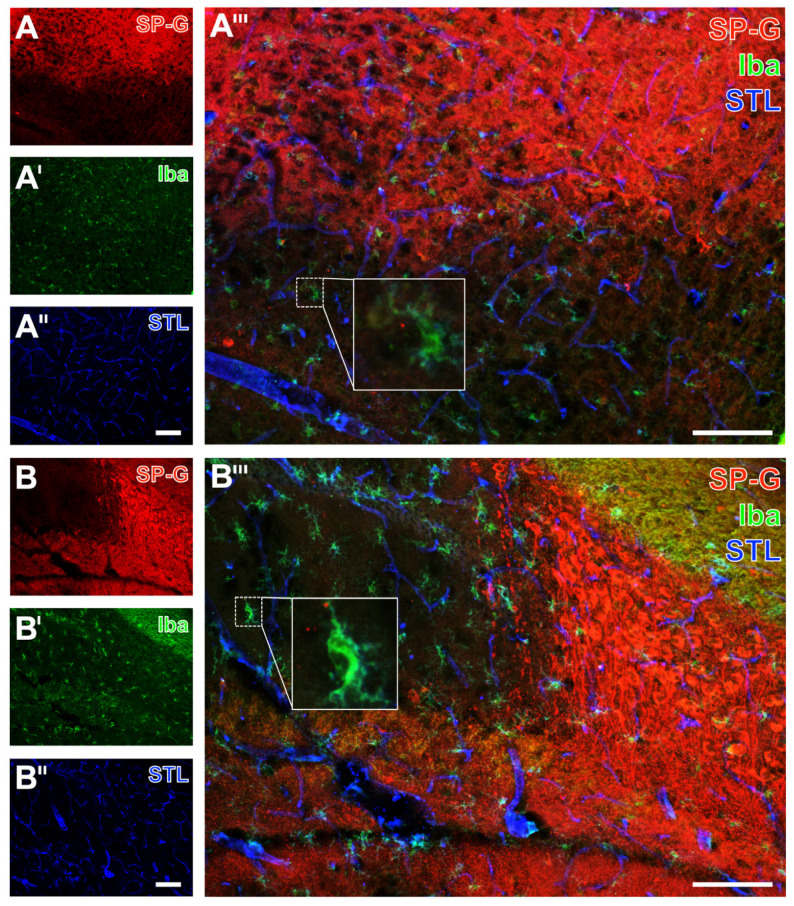
Microglia, surfactant protein-G (SP-G), and vessels after focal cerebral ischemia in mice. Triple fluorescence labeling of SP-G, ionized calcium-binding adapter molecule-1 (Iba) for microglia and endothelial lectin-binding sites (STL) visualizing the vasculature in the affected neocortex of a mouse subjected to 24 h of focal cerebral ischemia (**A**–**A’’’**). Exemplary combined immunofluorescence-based detection of SP-G, ionized calcium-binding adapter molecule-1 (Iba) for microglia, and lectin-based visualization of the vasculature (STL) in the hippocampus of a mouse subjected to 24 h of focal cerebral ischemia (**B**–**B’’’**). Scale bars: **A’’** (also valid for **A** and **A’**) = 100 μm, **A’’’** = 100 μm, **B’’** (also valid for **B** and **B’**) = 100 μm, **B’’’** = 100 μm.

As exemplarily shown in a mouse with 24 h of ischemia, immunosignals of SP-G and the neuron-associated NeuN were found to decrease in the ischemia-affected area (left side in Figure 6A,A’,A’’’). The band with a slightly increased SP-G signal, which was regularly observed to surround the area of maximum ischemia in the striatum (Figure 6A’’’), was characterized by a gradually reduced NeuN-ir. As observed in a mouse with 72 h of ischemia, the oligodendroglial CNP-ir increased in the ischemia-affected neocortex, which was accompanied by a reduced signal of SP-G (left side in Figure 6B,B’,B’’’) and an increased signal of collagen IV (left side Figure 6B’’,B’’’). Microglial Iba-ir was homogeneously distributed in the ischemia-affected neocortex (Figure 7A’) and in the hippocampus (Figure 7B’). As expected, morphological changes of detected microglia towards a ramified appearance became visible in areas with ischemic affection (bottom in Figure 7A’’’, and top left in Figure 7B’’’).

**Figure 8:**
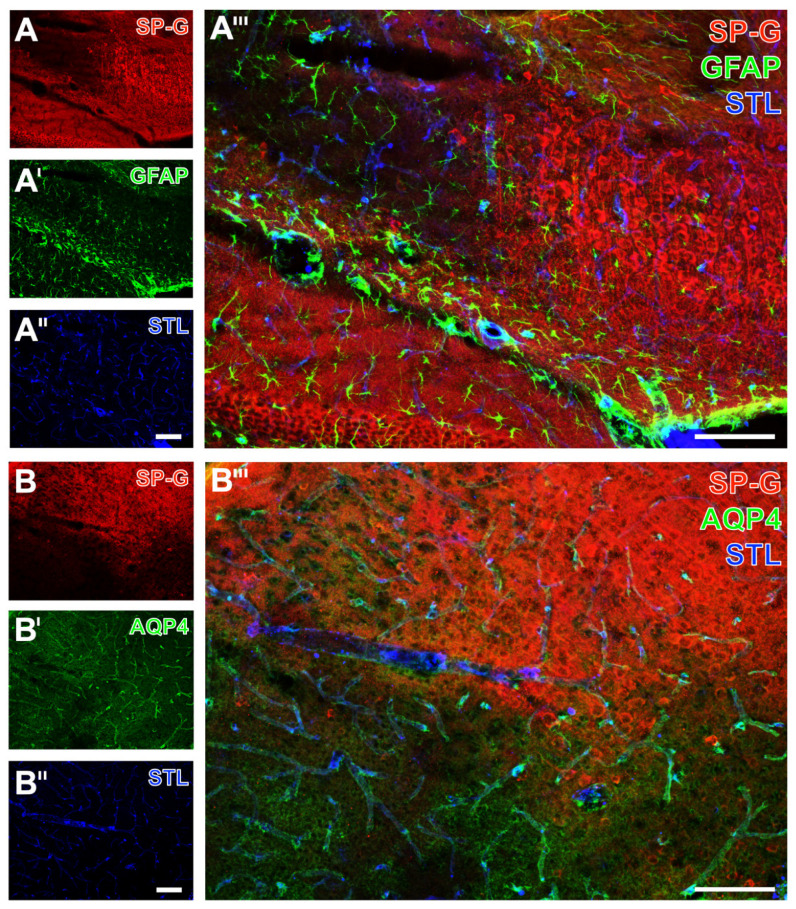
Astroglia, surfactant protein-G (SP-G), and vessels after focal cerebral ischemia in mice. Exemplary triple fluorescence labeling of SP-G, glial fibrillary acidic protein (GFAP), and lectin-based visualization (STL) of the vasculature in a mouse 24 h after focal cerebral ischemia in the affected hippocampus (**A**–**A’’’**) and neocortex (**B**–**B’’’**). Scale bars: **A’’** (also valid for **A** and **A’**) = 100 μm, **A’’’** = 100 μm, **B’’** (also valid for **B** and **B’**) = 100 μm, **B’’’** = 100 μm.

Simultaneously to these morphological changed Iba signals, a reduced SP-G signal in both the neocortex (Figure 7A’’’) and the hippocampus (Figure 7B’’’) became visible, as exemplarily shown in a mouse subjected to 24 h of ischemia. In these regions, endothelia, as visualized by *Solanum tuberosum* lectin (STL, from potato), remained nearly unchanged merely with a thinner appearance of vessels towards the area with maximum ischemia (bottom in Figure 7A’’,A’’’, top left in Figure 7B’’,B’’’). The GFAP signal, which was used to visualize astrocytes, was not distinctly altered in ischemic areas that were characterized by a decreased SP-G-ir. At least in part, the GFAP-ir was slightly changed towards a thinned appearance of visualized astrocytes due to ischemia, as exemplarily shown in the hippocampus of a mouse after 24 h of ischemia (top left in Figure 8A’,A’’’). In the neocortex, a homogeneous pattern of AQP4 was found at the ischemic border zone, exemplarily visualized in a mouse with 24 h of ischemia (Figure 8B’,B’’’). Here, the AQP4 signal appeared diffusely arranged but also associated with the vasculature, likely visualizing astroglial end-feet. 

Overall, the applied multiple fluorescence labeling in mice revealed several regional associations between SP-G and elements of the ischemia-affected NVU. In detail, decreasing immunosignals of neurons came along with a decreased signal of SP-G and an increased signal of collagen IV. Despite the strong regional association of changed SP-G-ir and vascular elements, a definite co-localization was not seen. Concerning glial elements, the ischemia-associated reduction of the SP-G signal co-occurred with an increased signal of oligodendroglial CNP and morphological changes of micro- and astroglia.

### 2.3. SP-G and the Associated Vasculature in Ischemia-Affected Brains from Rat and Sheep

To confirm the observed changes of immunosignals originating from SP-G as well as the regionally associated vasculature and NVU elements in other models of focal cerebral ischemia, selected brain sections from rats and sheep were analyzed. 

**Figure 9:**
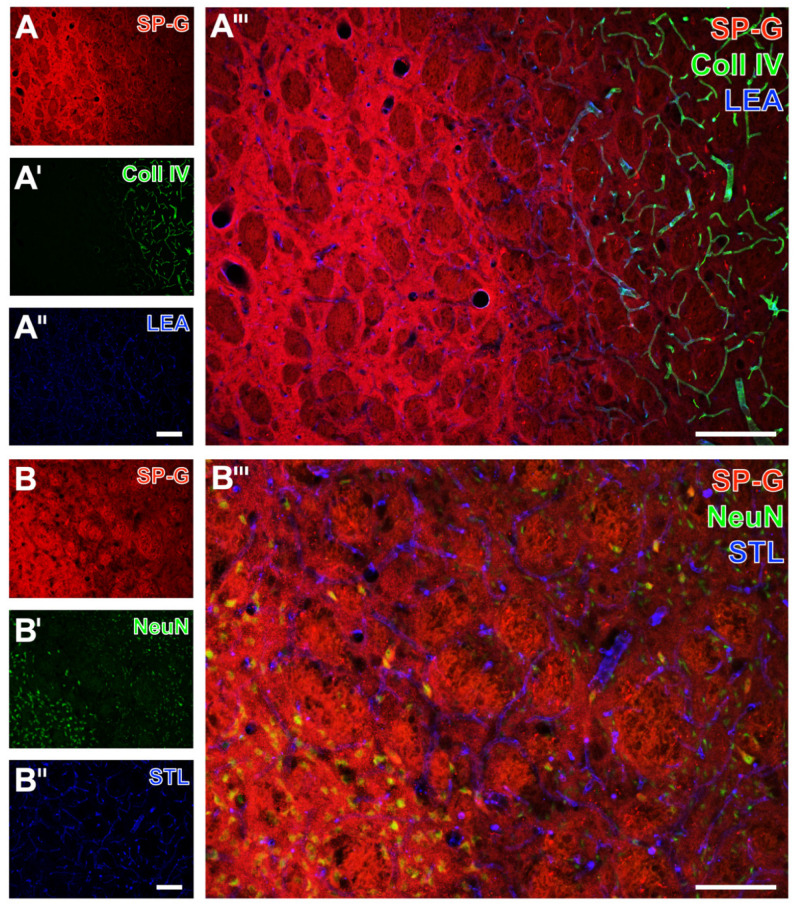
Regional associations of surfactant protein-G (SP-G), vascular elements, and neurons after focal cerebral ischemia in rats. Representative triple fluorescence labeling of SP-G, collagen (Coll) IV, and vascular binding sites for *Solanum tuberosum* lectin (STL) in the affected striatum after 24 h of focal cerebral ischemia (**A**–**A’’’**). Exemplary combined fluorescence-based detection of SP-G, the neuronal marker NeuN and STL-stained endothelia in the ischemia-affected striatum of a rat subjected to 24 h of focal cerebral ischemia (**B**–**B’’’**). Scale bars: **A’’** (also valid for **A** and **A’**) = 200 μm, **A’’’** = 200 μm, **B’’** (also valid for **B** and **B’**) = 100 μm, **B’’’** = 100 μm.

After 24 h of focal cerebral ischemia by an embolic model in rats, SP-G and collagen IV were similarly altered as in mice, while LEA and STL staining remained largely unaffected. In detail, the ischemia-affected striatum was characterized by a diminished SP-G-ir (right part in Figure 9A,A’’’) and a simultaneously increased collagen IV-ir (Figure 9A’,A’’’), while the stainability of endothelia with STL remained nearly unchanged (Figure 9A’’,A’’’). Concomitant detection of NeuN and SP-G revealed diminished neuronal staining in the ischemia-affected striatum, which was accompanied by a reduced SP-G signal (Figure 9B,B’,B’’’). 

**Figure 10:**
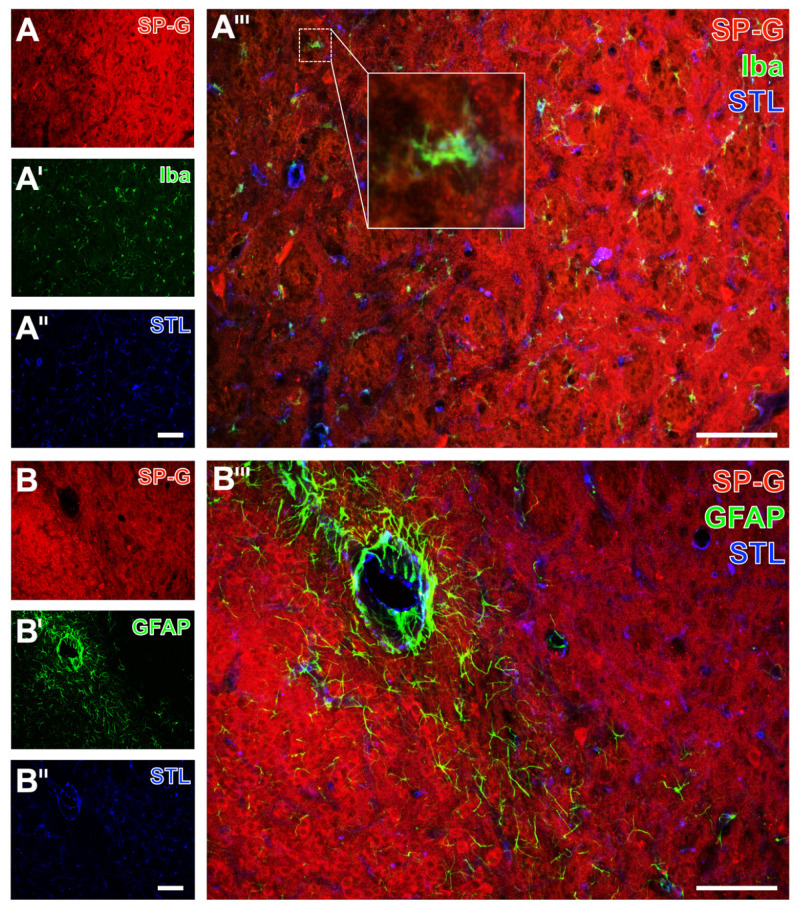
Regional associations of surfactant protein-G (SP-G), vascular and glial elements after focal cerebral ischemia in the striatum of rats. Triple fluorescence labeling of SP-G, ionized calcium-binding adapter molecule-1 (Iba) for microglia and vascular STL-binding sites in a rat 24 h after focal cerebral ischemia (**A**–**A’’’**). Immunolabeling of SP-G and astroglial GFAP combined with vascular STL-staining (**B**–**B’’’**). Scale bars: **A’’** (also valid for **A** and **A’**) = 100 μm, **A’’’** = 100 μm, **B’’** (also valid for **B** and **B’**) = 100 μm, **B’’’** = 100 μm.

Referring to the glial part of the NVU, concomitant staining of SP-G and Iba as a microglial marker showed homogeneously distributed microglia in the ischemia-affected striatum, as exemplarily shown in a rat with 24 h of focal cerebral ischemia. Similar to the findings in mice, morphological changes of microglia towards a ramified appearance in the ischemia-affected area became visible (left part in Figure 10A’,A’’’), which was characterized by a concomitantly reduced SP-G signal (left part in Figure 10A,A’’’). Regarding astrocytes, GFAP-ir appeared slightly decreased in the ischemic striatum (upper right part in Figure 10B’,B’’’), while SP-G decreased in the same region (upper right part in Figure 10B,B’’’). 

**Figure 11:**
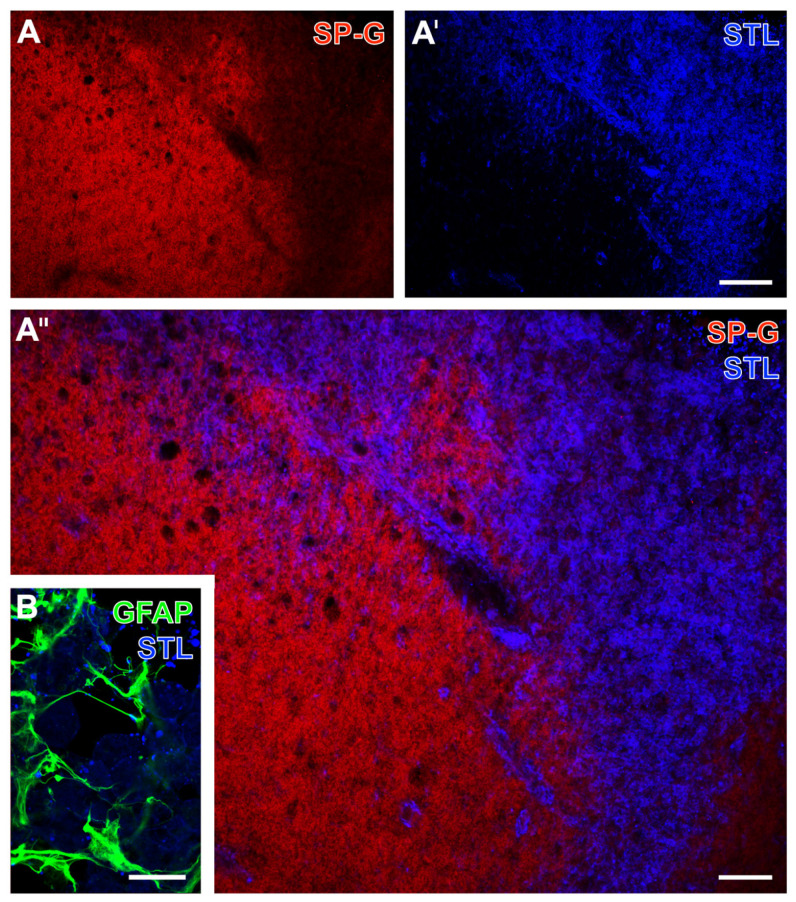
Spatial relationships between surfactant protein-G (SP-G), vascular and astroglial elements after focal cerebral ischemia in sheep. Representative double fluorescence labeling of SP-G and lectin-based visualization (STL) of the vasculature and inflammatory cells at the ischemic border zone after two weeks of focal cerebral ischemia (**A**–**A’’**). Additionally, astroglial GFAP in terms of a glial scar and STL-binding sites of the vasculature and inflammatory cells at the ischemic border zone, captured by laser-scanning microscopy (**B**). Scale bars: **A’** (also valid for **A**) = 200 μm, **A’’** = 100 μm, **B** = 25 μm.

Selected brain sections from sheep, which were subjected to focal cerebral ischemia by a coagulation-based model two weeks before, were applied to immunolabeling of SP-G with a lectin-based visualization of vascular elements. Although the relation of SP-G-ir to ischemia was not as clear as seen in the rodent models, a largely decreased SP-G-immunosignal was observed due to ischemia (top right part in Figure 11A,A’’). Starting at the border zone, an increased STL-ir was detectable towards ischemia visualizing vascular elements and likely an accumulation of inflammatory cells (Figure 11A’,A’’). Additional immunolabeling of GFAP together with STL indicated a scar-like formation of astrocytes at the ischemic border zone as a region with an already existing STL-ir (Figure 11B). 

Overall, findings in rats and sheep regarding SP-G as well as the associated vasculature and other neurovascular elements largely confirmed the observations in mice. Regional associations were seen in terms of a decreased signal of SP-G in areas of ischemic affection, while signals associated with the vasculature and notably collagen IV increased.

## 3. Discussion

The present study was intended as the first spatio-temporal characterization of SP-G and the associated vasculature as well as other elements of the NVU in experimental stroke. This approach was based on multiple fluorescence labeling, allowing the simultaneous visualization of two or three components and thus exploring regional and temporal allocations. Existing recommendations regarding experimental stroke research highlighted the role of applied models and the involvement of more than a single species to overcome translational hurdles [30]. Therefore, a highly standardized model, i.e., the filament-based model in mice, served for screening experiments and quantifications in the present study [31]. Further, an embolic model in rats and a coagulation-based model in sheep were included for confirmatory analyses. From a translational perspective, the applied filament-based model nicely reflects the clinical situation of endovascular-based recanalization approaches [32]. In contrast, the embolic model closely mimics a significant cause of stroke in humans [31].

This study revealed consistently decreased immunosignals of SP-G in areas of focal cerebral ischemia in mice, rats, and sheep at different time points after ischemia. Quantifications in mice subjected to 4 h and 24 h of ischemia showed a gradual reduction along the ischemia-affected neocortex towards the area with maximum ischemia. Further, ischemia-affected subcortical regions were characterized by a significantly decreased SP-G-ir. At the same time, a band-like formation with a slightly increased signal became visible at the border zone of subcortical ischemia. Regarding morphological aspects, in areas of ischemia, SP-G signals appeared as local accumulations with a possible cellular association but also in a weaker and diffusely distributed formation. Considering a recent study that described neuronal SP-G-ir in forebrain regions of the unaltered mouse’s brain [28], the here observed disintegration of the signal towards a much weaker and more diffusely arranged appearance might indicate the degradation of SP-G in the setting of ischemia. Concomitant visualization of SP-G-ir and vascular collagen IV-immunopositive structures robustly showed inverse immunosignals in areas with ischemic affection, which means a significant decrease of SP-G and an increase of collagen IV-ir. Immunosignals of both SP-G and collagen IV were affected most clearly in the area with maximum ischemia and normalized gradually towards the non-affected area along the neocortex in mice. The observed pattern of collagen IV-ir confirmed an earlier report, which also described a gradually increased affection towards the area with maximum ischemia in the neocortex [33]. In the present study, a close association was visible when focusing on the regional arrangement of SP-G and the vasculature. However, a definite co-localization of SP-G and collagen IV was not seen, at least when using an immunofluorescence-based setup with subsequent validation by laser-scanning microscopy. At first sight, this observation contrasts with an earlier report describing a co-localization of SP-G and vascular markers in rat brains [29]. However, these different observations likely resulted from methodological differences. The study from Krause et al. applied AQP4 and the platelet endothelial cell adhesion molecule-1 (CD31) to detect vascular elements generally [29]. Compared to the lectin- and collagen IV-based visualization of the vasculature in the present study, AQP4 and CD31 also cover functional aspects such as swelling of the astrocyte’s end-feet and an activated binding site for platelets. This view is supported by a very recent study involving brain sections of naive mice, which has shown a missing overlap of SP-G and lectin-stained vascular endothelia [28].

While analyzing non-vascular elements of the NVU along with the decreasing signal of SP-G in areas of ischemic affection, decreased signals of neuronal NeuN and increased signals of oligodendroglial CNP were found. These affections of neurons and oligodendrocytes due to ischemia confirmed previous reports, which have applied immunofluorescence-based techniques to explore ischemic consequences to NVU components (e.g., [6,34]). Regarding microglia and astrocytes, visualized by Iba, GFAP, and AQP4, the observed ischemia-associated alterations were in line with earlier reports exploring cellular changes due to ischemia (e.g., [6,7]). To address components not typically covered by the NVU [4], the present study also included fibronectin, which was seen as a linking protein between the vascular part of the NVU and the extracellular matrix [33,35]). Thereby, a decreased signal of fibronectin was observed in parallel with a decreased SP-G signal in areas with maximum ischemia, indicating at least a regional association between SP-G and elements beyond the NVU.

Collectively, a regional association between SP-G and vasculature elements of the NVU in terms of inverse patterns of SP-G- and collagen IV-ir was consistently found at different time points after experimental focal cerebral ischemia. Further, the decrease of SP-G-ir within areas of maximum ischemic affection co-occurred with either altered or at least morphologically changed immunosignals of non-vascular, i.e., cellular elements of the NVU. These findings strongly stimulate a discussion regarding the functional role of SP-G in the context of focal cerebral ischemia. From the variety of pathophysiological mechanisms contributing to ischemia-associated tissue damage [3], at first sight, SP-G seems unlikely to be involved in inflammatory or immune-related processes as regulatory domains were not detected in structural analyses of SP-G [27]. On the other hand, Krause et al. described increased levels of SP-G in the cerebrospinal fluid of patients with bacterial infection of the central nervous system [29], which ultimately leads to the perspective that involvement in inflammatory processes is possible. Whether other mechanisms that are known to contribute to brain damage in the early stage of ischemia [3], e.g., excitotoxicity, might be linked to SP-G remains speculative at this time. However, the observed simultaneous alteration of SP-G and vascular elements supports a theory of an involvement in fluid balance regulations and edema formation after stroke. This view is also supported by the time frame in which the here reported immunosignal alterations became detectable. In detail, previous studies revealed stroke-related affections on the vasculature’s integrity very early after ischemia onset, i.e., within hours [36], which is maintained over days [37] or even weeks [38]. Thereby, AQP4, located at the astrocyte’s end-feet [39], was discussed to have a pivotal role in edema formation after stroke [14,19,20]. Regulatory properties of AQP4 relate to the blood-brain barrier [10,21], the influx of cerebrospinal fluid [13], and the glymphatic system [39].

This study has some limitations: First, given the larger family of SPs, analyses were limited to SP-G, which was chosen as the recent literature suggested a regional association between vascular and non-vascular elements of the NVU [28,29]. Second, although a couple of vascular markers were applied in the present study, alternative approaches to visualize the vasculature remain manifold. Therefore, using other markers than those applied here could provide more insight into regional associations with SP-G and its cellular sources. Third, efforts were made to characterize SP-G and vascular elements at different time points, though 4, 24, and 72 h were chosen to allow comparison with other studies. Unfortunately, quantifications were limited to 4 h and 24 h of ischemia in mice due to limited brain tissues. This limitation seems relevant, especially for analyses of brain sections from sheep with a single and relatively late time point. Generally, quantitative analyses at later observation time points are difficult in experimental stroke. Models with rather small cortical infarcts and only minor neurobehavioral affections might be useful but appear clinically less relevant. Fourth, immunofluorescence-based techniques generally bear the risk of misinterpretation regarding functional aspects. In detail, changed signals might be caused by degenerating processes that result in more accessible free binding sites for the applied antibodies and thus an increased immunosignal. Therefore, this study was designed to strictly follow a descriptive way and focused on changed immunolabeling, which indicates alterations of cellular or non-cellular structures, regardless of their functional relevance. This study thus does not provide causal relationships between SP-G and cellular or non-cellular affections typically occurring during ischemia, which need to be addressed in future studies. 

Despite the given limitations, this study, for the first time, provides a spatio-temporal characterization of SP-G and its regional associations with vascular and other elements of the NVU after experimental focal cerebral ischemia. Although SP-G was not found to definitely overlap with vascular elements, inversely altered immunosignals of SP-G and notably collagen IV were robustly observed within ischemia-affected areas. Regarding non-vascular elements within the NVU, the ischemia-related decrease of the SP-G signal co-occurred with decreased signals of neurons, increased signals of oligodendrocytes, and morphologically changed micro- and astroglia. Although these findings are rather descriptive, they may stimulate the discussion about SP-G’s role within the ischemia-affected NVU, which might be linked to the integrity of the vasculature and thus to fluid balance regulations in stroke. 

## 4. Materials and Methods

### 4.1. Study Design and Content

Multiple immunohistochemical analyses were performed to characterize SP-G together with regionally associated vascular structures and other NVU elements in brain tissues affected by focal cerebral ischemia. The applied ischemia models in mice, rats, and sheep are described below. For qualitative analyses, brains from mice with an ischemia duration of 4 h (*n* = 7), 24 h (*n* = 8), and 72 h (*n* = 2), and brains from rats with an ischemia duration of 24 h (*n* = 4), and brain tissues from sheep affected by ischemia two weeks before (*n* = 2) were used. For quantitative analyses, selected brain sections from mice with an ischemia duration of 4 h (*n* = 6) and 24 h (*n* = 5) were used. However, the available material from mice subjected to 72 h of ischemia was not sufficient to apply robust quantitative analyses.

Animal experiments were carried out following the European Union Directive 2010/63/EU and the German guideline for the care and use of laboratory animals. The Regierungspräsidium Leipzig as the local authority approved all experiments (reference numbers: TVV 02/17, and TVV 56/15). Reporting followed the ARRIVE criteria for experimental research [40]. 

### 4.2. Experimental Focal Cerebral Ischemia in Mice, Rats, and Sheep

C57Bl/6J mouse adult males with an approximate body weight of 25 g (bred by Charles River, Sulzfeld, Germany) were subjected to permanent right-sided middle cerebral artery occlusion by a filament-based model, described initially by Longa et al. [41] with minor modifications [42]. Briefly, right-sided cervical arteries were carefully exposed using an operation microscope (Zeiss, Oberkochen, Germany). A standardized silicon-coated 6-0 monofilament (Doccol Corporation, Redlands, CA, USA) was inserted into the internal carotid artery and moved forward until bending was observed or resistance was felt (approximately 9 mm from carotid bifurcation). The filament was left in place while the skin was closed with a surgical suture. Adult male Wistar rats with an approximate body weight of 300 g (bred by Charles River) were subjected to permanent right-sided middle cerebral artery occlusion by a thromboembolic model, described initially by Zhang et al. [43] with minor modifications [6]. Briefly, right-sided cervical arteries were carefully exposed with an operation microscope (Zeiss). A polyethylene (PE) tube with a tapered end was introduced into the external carotid artery and moved forward through the internal carotid artery (approximately 16 mm from carotid bifurcation). At this position, a clot originating from rat blood was injected. The catheter was then removed, and the skin was closed with a surgical suture. 

For surgical procedures in mice and rats, anesthesia was performed with about 2–2.5 % isoflurane (Isofluran Baxter, Baxter, Unterschleißheim, Germany) in a mixture of 70% N_2_O/30% O_2_, applicated with a commercial vaporizer (VIP 3000, Matrix, New York, NY, USA). A thermostatically controlled warming pad with a rectal probe (Fine Science Tools, Heidelberg, Germany) was used to prevent anesthesia-associated cooling during surgical procedures, allowing a stable body temperature at around 37 °C. Rodents received a complex pain medication including lidocaine (e.g., Licain, DeltaSelect, Dreieich, Germany), meloxicam (e.g., Metacam, Boehringer Ingelheim Vetmedica, Ingelheim, Germany), and metamizole (e.g., Novaminsulfon-ratiopharm, Ratiopharm, Ulm, Germany), respectively. A relevant neurobehavioral deficit in terms of at least two points on the Menzies score (ranging from 0, no deficit, to 4, spontaneous contralateral circling; [44]) indicated successful focal cerebral ischemia. Pre-defined time points for histochemical evaluation were 4, 24, and 72 h in mice and 24 h in rats, each from ischemia induction.

Adult male sheep (hornless Merino) with an approximate body weight of 70 kg (provided by the Veterinary Faculty of Leipzig University, Lehr- und Versuchsgut Leipzig, Germany) were subjected to left-side middle cerebral artery occlusion by a surgical procedure, described by Nitzsche et al. [45] and Boltze et al. [46]. Briefly, the temporal bone was carefully exposed, followed by a trepanation with a 6 mm barrel burr at 10,000 rpm (Aesculap micro speed uni, Scil Animal Care Company, Viernheim, Germany). After cutting the dura mater, the middle cerebral artery was exposed and occluded at the distal M1 segment by electrosurgical coagulation using a neurosurgical bipolar forceps (ME 411, KLS Martin, Tuttlingen, Germany). Finally, the dura mater was repositioned, while muscles and the skin were closed with surgical sutures. For the surgical procedure, anesthesia was performed by intravenous injection of ketamine (4 mg/kg body weight; Ketamin, Medistar, Holzwicke, Germany), xylazine (0.1 mg/kg body weight; Xylazin, Ceva Sante Animal, Düsseldorf, Germany), and diazepam (0.2 mg/kg body weight; Temmler Pharma, Marburg, Germany). During surgery, mechanical ventilation was performed with 2% isoflurane and 40% oxygen (Primus, Dräger, Lübeck, Germany). At the end of the surgery, sheep were treated with the antibiotic enrofloxacin (5% Baytril, Bayer, Leverkusen, Germany), while butorphanol (Alvegesic 1%; CP-pharm, Burgdorf, Germany) was used for pain medication. Successful focal cerebral ischemia was confirmed by magnetic resonance imaging. The pre-defined time point for histochemical evaluation was two weeks from ischemia induction. 

### 4.3. Tissue Preparation and Fluorescence Labeling

At the end of the observation period (4, 24, or 72 h in rodents, and two weeks in sheep), animals were sacrificed and perfused with saline, followed by perfusion with 4 % phosphate-buffered paraformaldehyde (PFA). All PFA-fixed brains from rodents and sheep were equilibrated with 30 % phosphate-buffered sucrose. Mouse and rat forebrains were cut with a freezing microtome (Leica SM 2000R, Leica Biosystems, Wetzlar, Germany), while 10 series of 30 µm-thick sections were collected. Blocs from sheep forebrains were cut at 40 µm thickness by applying a freezing microtome (Microm HM 430, Thermo Fisher Scientific, Waltham, MA, USA). Sections of rodents and sheep were stored at 4 °C in 0.1 M Tris-buffered saline, pH 7.4 (TBS), containing sodium azide.

Histochemical procedures started with an extensive rinsing of sections in TBS followed by blocking non-specific binding sites with 5 % normal donkey serum in TBS containing 0.3% Triton X-100 (NDS-TBS-T) for one hour. Next, sections were processed with mixtures of three primary antibodies from different host species or two primary antibodies and a biotinylated lectin, diluted in NDS-TBS-T (details in Table 1). Applied biotinylated lectins were LEA (from tomato) and STL (from potato), which were provided by Vector Laboratories (Burlingame, CA, USA) and used at 20 µg/mL each. After an incubation of 20 h, the sections were rinsed in TBS and then reacted with mixtures of appropriated fluochromated immunoreagens, e.g., highly purified donkey antibodies and streptavidin conjugates [Dianova, Hamburg, Germany; 20 µg/mL TBS containing 2% bovine serum albumin (TBS-BSA)]. The first set of ischemia-affected sections from mice was applied to the immunodetection of endogenous albumin by green fluorescent carbocyanine (Cy)2, SP-G by red fluorescent Cy3, and LEA-counterstaining by Cy5 (color-coded in blue). Further mouse sections underwent labeling of SP-G and fibronectin in combination with the visualization of LEA-binding sites. 

For quantitative analyses, serial sections from mice were applied to Cy3-labeling of SP-G and Cy2-staining with sheep-anti-collagen IV. For additional qualitative analyses, mouse and rat sections were primarily reacted with mixtures of anti-SP-G, biotinylated STL, and guinea pig antibodies directed against diverse NVU components, including NeuN, CNP, Iba, GFAP, and AQP4. Immunoreactivities and lectin-binding sites were visualized using a cocktail of Cy3-donkey-anti-rabbit IgG, Cy2-donkey-anti-guinea pig IgG, and Cy5-streptavidin. Infarcted brain tissue from sheep was first reacted with mixtures of rabbit-anti-SP-G, biotinylated STL, and finally, guinea pig antibodies directed against GFAP. The markers were then visualized by Cy3- and Cy2-tagged secondary antibodies and Cy5-streptavidin. 

In control experiments, the primary antibodies and biotinylated lectins were omitted resulting in the absence of biological signals.

Free-floating sections were extensively rinsed in TBS and briefly in distilled water prior to mounting onto glass slides. Finally, air-dried sections were coverslipped with Entellan in toluene (Merck, Darmstadt, Germany).

### 4.4. Microscopy and Processing of Images

An Axioplan fluorescence microscope (Zeiss) served for the first screenings. Micrographs on selected brain regions and different magnifications were captured with the fluorescence microscope Biorevo BZ-9000 (Keyence, Neu-Isenburg, Germany) and the confocal laser-scanning microscope LSM 880 (Zeiss). Panels of micrographs were generated with Microsoft PowerPoint (Office 365, Version 2021; Microsoft Corp., Redmond, WA, USA) and Adobe Photoshop CS5 Extended (Version 12.0 ×64; Adobe Systems Incorporated, San Jose, CA, USA). If necessary, the brightness and contrast of micrographs were slightly adjusted without the deletion or creation of signals.

### 4.5. Quantification of Fluorescence Signals

Quantitative analyses of immunofluorescence signals of SP-G and regionally associated collagen IV were usually based on 5 brain sections from each mouse. Selection criteria included an ischemic affection visualized by regional changes of fluorescence signals at the right hemisphere, as known from own earlier studies using the same ischemia model (e.g., Mages et al., 2018). A further requirement was the absence of tissue damage in large parts of the ischemia-affected hemisphere that prevents fluorescence-based analyses. The applied selection process allowed the inclusion of 6 out of the available 7 mice with an ischemia duration of 4 h and 5 out of the available 8 mice with an ischemia duration of 24 h, while exclusion was necessary due to lack of injury in the areas planned for quantification. For quantification, 7 ROIs were arranged at the ischemia-affected hemisphere. In detail, 6 ROIs were positioned along the neocortex covering the ischemic border zone with the most evident changes of the SP-G immunosignal between ROIs 3 and 4. A further ROI was placed in the subcortex, i.e., the striatum. To allow inter-hemispheric comparison, these 7 ROIs were mirrored in the non-affected hemisphere, resulting in an overall number of 14 ROIs in each section. Micrographs of each ROI were captured with a 40× objective on the Biorevo microscope (Keyence, Osaka, Japan). To avoid overexposure and to enable inter-hemispheric comparison, the exposure time was adjusted at the level of each section. This technique usually resulted in 70 micrographs from each animal. Due to porous infarct material in a few mice, the most lateral ROIs of the neocortex were sometimes not obtainable, and in one mouse, only 4 sections were available. Overall, 738 micrographs were captured from 11 mice, which were included in quantitative analyses.

Immunofluorescence signals of SP-G were assessed by their mean values in the obtained ROI using ImageJ (National Institutes of Health, Bethesda, MD, USA). For collagen IV, the maximum immunosignal intensity was used to consider the strongly vessel-associated signal with a visually darker background on the affected hemisphere compared to the contralateral side. Finally, rounded mean values of the obtained fluorescence signals of SP-G and collagen IV were calculated for each ROI along the available brain sections at the level of each animal. Further calculations included an interhemispheric comparison with reference to ROIs along the ischemic border zone in the neocortex and the striatum. These calculations were done for both the overall sample and the two available time points (i.e., 4 h and 24 h) after ischemia induction.

### 4.6. Statistical Analyses

The SPSS software package version 27 (IBM SPSS Statistics for Windows, IBM Corp., Armonk, NY, USA) was applied for descriptive analyses and testing regarding the statistical significance between groups. Due to the relatively small sample size, non-parametric testing was necessary, which was realized by the Wilcoxon test added by the Bonferroni–Holm correction to consider multiple testing. Further, Pearson correlation coefficients were used to explore statistical relationships between parameters. Generally, a value of p < 0.05 was regarded as statistically significant. 

## Figures and Tables

**Table 1:** Applied primary antibodies.

Marker	Host Species	Supplier	Product Number	Dilution	Fluoro-phor
Albumin	Sheep	AbD Serotec, Oxford, UK	0220-2424	1:500	Cy2
Aquaporin 4	Guinea pig	Synaptic Systems, Göttingen, Germany	429004	1:200	Cy2
CNP	Guinea pig	Synaptic Systems	355004	1:200	Cy2
Collagen IV	Goat	Merck Millipore; Billerica, MA, USA	AB749	1:100	Alexa Fluor647
Fibronectin	Sheep	Bio-Techne; Wies-baden, Germany *	AF1918	1:100	Cy2
GFAP	Guinea pig	Synaptic Systems	173004	1:200	Cy2
Iba	Guinea pig	Synaptic Systems	234004	1:100	Cy2
NeuN	Guinea pig	Synaptic Systems	266004	1:200	Cy2
SP-G	Rabbit	Hölzel; Cologne, Germany **	PAD755 Hu01	1:70 ***	Cy3

*: Provider for R&D Systems; Minneapolis, MN; USA. **: Supplier for Cloud-Clone; Katy, TX, USA; ***: = 10 µg/mL. Abbreviations: CNP: 2′,3′ cyclic nucleotide phosphodiesterase; GFAP: glial fibrillary acidic protein; Iba: ionized calcium-binding adapter molecule 1; NeuN: neuronal nuclei; SP-G: surfactant protein-G.

## Data Availability

Data underlying this article will be made available by the authors upon reasonable request.
